# Supplementary material for: Lipid mediated plant immunity in susceptible and tolerant soybean cultivars in response to Phytophthora sojae colonization and infection
Source: BMC Plant Biol. 2024 Mar 1;24:154. doi: 10.1186/s12870-024-04808-z (PMC10905861; doi:10.1186/s12870-024-04808-z)
Supplement: Supplementary file 1 — Supplementary Material 1. [file 12870_2024_4808_MOESM1_ESM.docx]

**Additional file 1: Table S1.** Uniquely changed lipids for each comparison.

| Tissue | Contrast | Lipid |
| --- | --- | --- |
| Root | ORC_ORI  ORC_ORI  ORC_ORI  ORC_ORI  ORC_ORI  ORC_ORI  CRC_CRI  CRC_CRI  ORI_CRI  ORI_CRI  ORI_CRI  ORI_CRI  ORI_CRI | TG(20:1/18:1/18:2)  TG(18:1/18:1/18:1)  TG(8:0/8:0/8:0)  TG(18:0/16:0/18:1)  TG(16:0/18:3/18:3)  TG(16:0/16:0/18:3)  TG(18:4/11:3/12:4)  DG(18:0/18:0)  TG(18:3/18:2/23:0)  TG(10:0/10:0/10:0)  DG(18:3/18:3)  DG(16:0/18:3)  DG(22:0/18:2) |
| Stem | CSC_CSI  CSC_CSI  CSC_CSI  CSC_CSI  CSC_CSI  CSC_CSI | TG(12:0/12:0/12:0)  TG(16:0/16:0/18:2)  TG(10:0/10:0/14:1)  DG(20:0/22:0)  TG(10:0/10:0/14:0)  DG(24:0/18:2) |

This table lists lipids which are only significantly altered in the specified contrast when considering all root and stem treatment comparisons combined. All other lipids are not uniquely changed i.e., are significantly altered in more than 1 comparison. ORC = root of control susceptible soybean cultivar, ORI = root of inoculated susceptible soybean cultivar, CRC= root of control tolerant soybean cultivar, CRI = root of inoculated tolerant soybean cultivar, OSC = stem of control susceptible soybean cultivar, OSI = stem of inoculated susceptible soybean cultivar, CSC= stem of control tolerant soybean cultivar, CSI = stem of inoculated tolerant soybean cultivar.
