# Supplementary material for: Lipid mediated plant immunity in susceptible and tolerant soybean cultivars in response to Phytophthora sojae colonization and infection
Source: BMC Plant Biol. 2024 Mar 1;24:154. doi: 10.1186/s12870-024-04808-z (PMC10905861; doi:10.1186/s12870-024-04808-z)
Supplement: Supplementary file 2 — Supplementary Material 2. [file 12870_2024_4808_MOESM2_ESM.docx]

**Additional file 2: Table S2.** Characteristic product ions of some biomarkers.

|  | **Lipid class** | **Molecular species** | **m/z [M+NH4^+^]** | **M-FA1** | **M-FA2** | **M-FA3** |
| --- | --- | --- | --- | --- | --- | --- |
| **ORC vs. ORI** | TG 24:0  TG 52:1  TG 52:6  TG 50:3 | TG 8:0/8:0/8:0  TG 18:0/16:0/18:1  TG 16:0/18:3/18:3  TG 16:0/16:0/18:3 | 488.3946  878.8171  868.7389  846.7545 | 327.2535  577.5196  595.4726  573.4883 | 327.2535  605.5509  573.4883  573.4883 | 327.2535  579.5352  573.4883  551.5039 |
| **CRC vs. CRI** | TG 41:11  DG 36:0 | TG 18:4/11:3/12:4  DG 18:0/18:0 | 704.4885  642.6031 | 411.2535  341.3056 | 507.3474  341.3056 | 495.3474  607.5665 |
| **ORI vs. CRI** | TG 30:0  TG 59:5  DG 36:6  DG 34:3 | TG 10:0/10:0/10:0  TG 18:3/18:2/23:0  DG 18:3/18:3  DG 16:0/18:3 | 572.4885  968.8641  630.5092  608.5249 | 383.3161  673.6135  335.2586  313.2743 | 383.3161  671.5978  335.2586  335.2586 | 383.3161  597.4883  595.4726  573.4883 |
| **OSC vs. OSI** | DG 40:2 | DG 22:0/18:2 | 694.6344 | 337.2743 | 397.3682 | 659.5978 |
| **CSC vs. CSI** | TG 36:0  TG 50:2  TG 34:1  DG 42:0 | TG 12:0/12:0/12:0  TG 16:0/16:0/18:2  TG10:0/10:0/14:1  DG 20:0/22:0 | 656.5824  848.7702  626.5354  726.6970 | 439.3787  575.5039  437.3631  397.3682 | 439.3787  575.5039  437.3631  369.3369 | 439.3787  551.5039  383.3161  691.6604 |
| **OSI vs. CSI** | TG 34:0  DG 42:2 | TG 10:0/10:0/14:0  DG 24:0/18:2 | 628.5511  722.6657 | 439.3787  337.2743 | 439.3787  425.3995 | 383.3161  687.6291 |

This table demonstrated the characteristic product ions of some biomarkers differentiating the root and stem lipidomes of the soybean cultivars, identified from their lipid biochemical network (shown in Figures 10-12). ORC = root of control susceptible soybean cultivar, ORI = root of inoculated susceptible soybean cultivar, CRC= root of control tolerant soybean cultivar, CRI = root of inoculated tolerant soybean cultivar, OSC = stem of control susceptible soybean cultivar, OSI = stem of inoculated susceptible soybean cultivar, CSC= stem of control tolerant soybean cultivar, CSI = stem of inoculated tolerant soybean cultivar.
