# Supplementary material for: Lipid mediated plant immunity in susceptible and tolerant soybean cultivars in response to Phytophthora sojae colonization and infection
Source: BMC Plant Biol. 2024 Mar 1;24:154. doi: 10.1186/s12870-024-04808-z (PMC10905861; doi:10.1186/s12870-024-04808-z)
Supplement: Supplementary file 3 — Supplementary Material 3. [file 12870_2024_4808_MOESM3_ESM.docx]

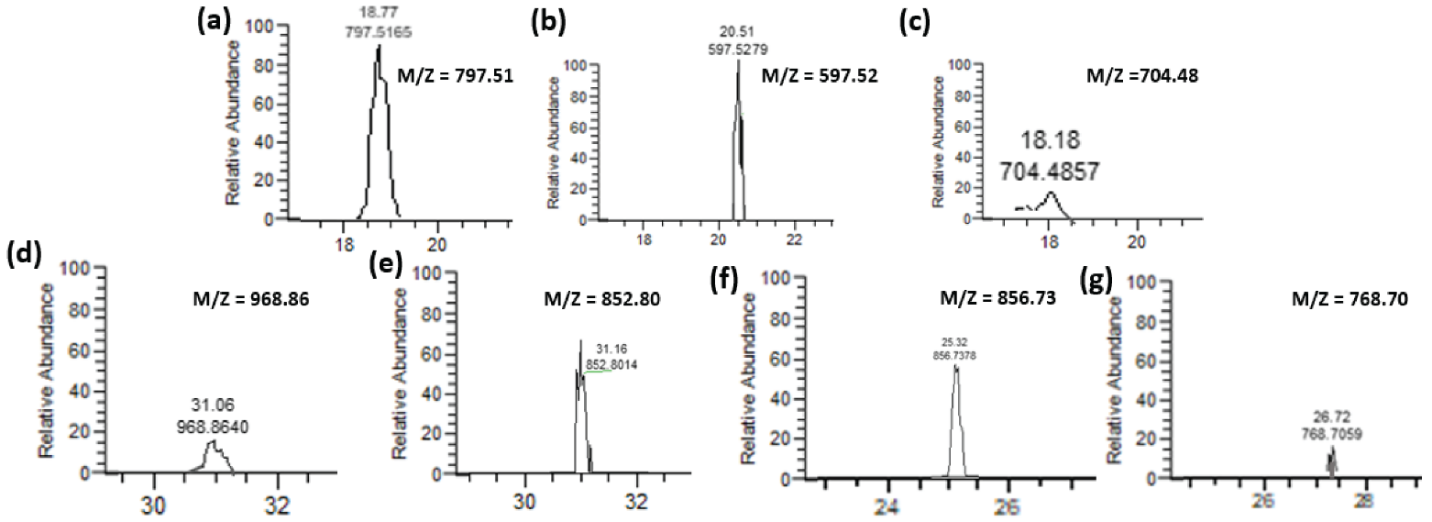


**Additional file 3: Fig. S1.** Extracted ion chromatogram (XIC) of odd chain FAs (a) *m/z* 7971.51 for PI18:/13:0 [M+H]^+^, (b) *m/z* 597.52 for DG15:0/16:0 [M+NH_4_]^+^, (c) *m/z* 704.48 for TG18:4/11:3/12:4 [M+NH_4_]^+^, (d) *m/z* 968.86 for TG18:3/18:2/23:0 [M+NH4]4, (e) *m/z* 852.80 for TG16:0/17:0/17:0 [M+NH4]+, (f) *m/z* 856.73 for TG15:0/18:2/18:3 [M+NH4]+ and (g) *m/z* 768.70 for TG15:0/14:0/15:0 precursor ions of the one phospholipid and six neutral lipids, identified in the positive ion mode; PI = phosphatidylinositol, DG = diacylglycerol, TG = triacylglycerol.
