# Supplementary figures and images for: Lipid mediated plant immunity in susceptible and tolerant soybean cultivars in response to Phytophthora sojae colonization and infection
Source: BMC Plant Biol. 2024 Mar 1;24:154. doi: 10.1186/s12870-024-04808-z (PMC10905861; doi:10.1186/s12870-024-04808-z)

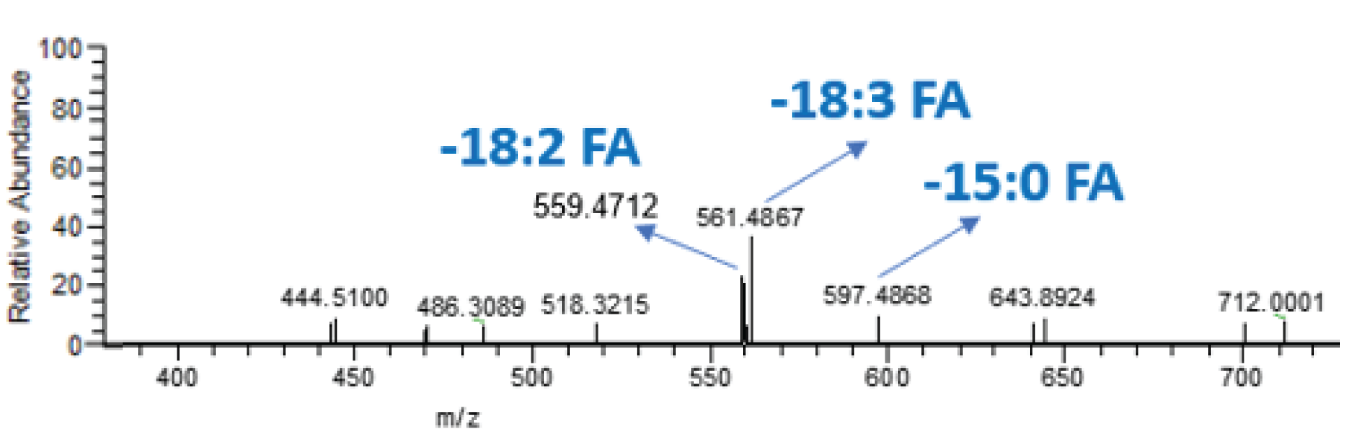


**Additional file 4: Fig. S2.** MS2 spectrum of *m/z* 856.73 representing TG 15:0/18:2/18:3 [M+NH4]+ identified in the positive ion mode.

Supplement: Supplementary file 4 — Supplementary Material 4. [file 12870_2024_4808_MOESM4_ESM.docx]
