# Supplementary material for: Lipid mediated plant immunity in susceptible and tolerant soybean cultivars in response to Phytophthora sojae colonization and infection
Source: BMC Plant Biol. 2024 Mar 1;24:154. doi: 10.1186/s12870-024-04808-z (PMC10905861; doi:10.1186/s12870-024-04808-z)
Supplement: Supplementary file 5 — Supplementary Material 5. [file 12870_2024_4808_MOESM5_ESM.docx]

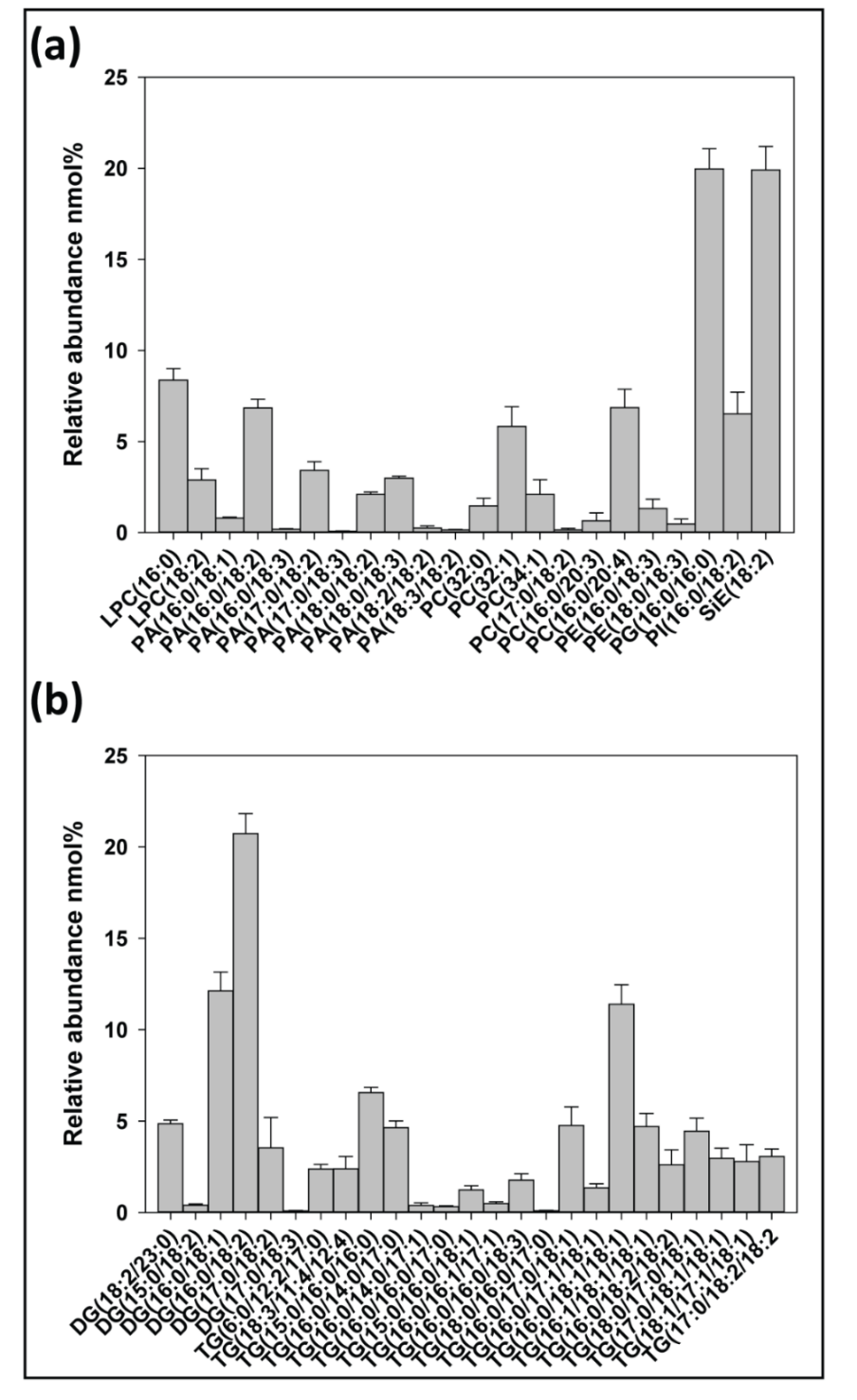


**Additional file 5: Fig. S3.** Membrane lipid and neutral lipid molecular species identified in mycelium of *P. sojae.* (a) Membrane lipid molecular species identified in mycelium of *P. sojae* and (b) Neutral lipid molecular species identified in mycelium of *P. sojae*.
