# Supplementary material for: Lipid mediated plant immunity in susceptible and tolerant soybean cultivars in response to Phytophthora sojae colonization and infection
Source: BMC Plant Biol. 2024 Mar 1;24:154. doi: 10.1186/s12870-024-04808-z (PMC10905861; doi:10.1186/s12870-024-04808-z)
Supplement: Supplementary file 6 — Supplementary Material 6. [file 12870_2024_4808_MOESM6_ESM.docx]

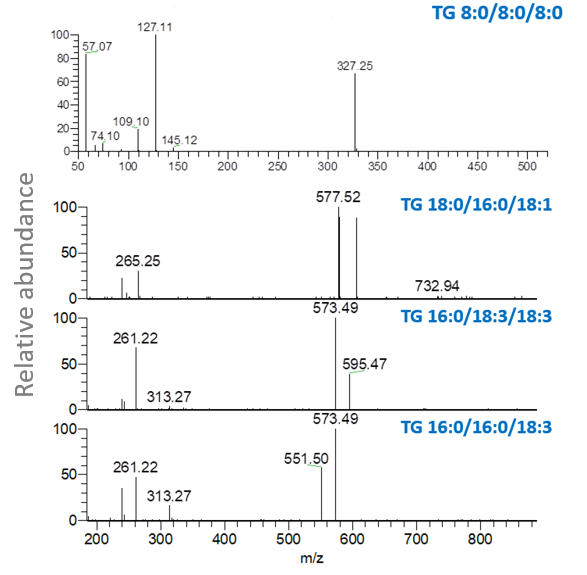


**Additional file 6: Fig. S4.** UHPLC-HRMS/MS mass spectra of TG 8:0/8:0/8:0, TG 18:0/16:0/18:1, TG 16:0/18:3/18:3 and TG16:0/16:0/18:3 which were unique biomarkers differentiating the ORC vs. ORI.
