# Supplementary material for: Lipid mediated plant immunity in susceptible and tolerant soybean cultivars in response to Phytophthora sojae colonization and infection
Source: BMC Plant Biol. 2024 Mar 1;24:154. doi: 10.1186/s12870-024-04808-z (PMC10905861; doi:10.1186/s12870-024-04808-z)
Supplement: Supplementary file 7 — Supplementary Material 7. [file 12870_2024_4808_MOESM7_ESM.docx]

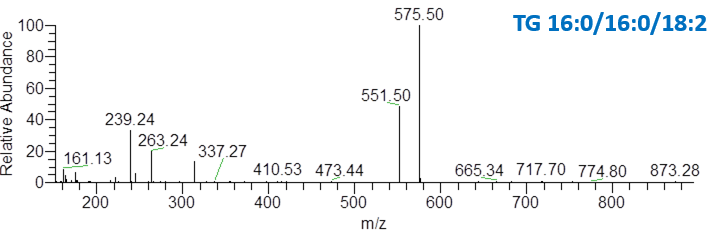


**Additional file 7: Fig. S5.** UHPLC-HRMS/MS mass spectrum of TG16:0/16:0/18:2 which was one of the unique biomarkers differentiating CSC vs. CSI.
