# Supplementary material for: Lipid mediated plant immunity in susceptible and tolerant soybean cultivars in response to Phytophthora sojae colonization and infection
Source: BMC Plant Biol. 2024 Mar 1;24:154. doi: 10.1186/s12870-024-04808-z (PMC10905861; doi:10.1186/s12870-024-04808-z)
Supplement: Supplementary file 8 — Supplementary Material 8. [file 12870_2024_4808_MOESM8_ESM.docx]

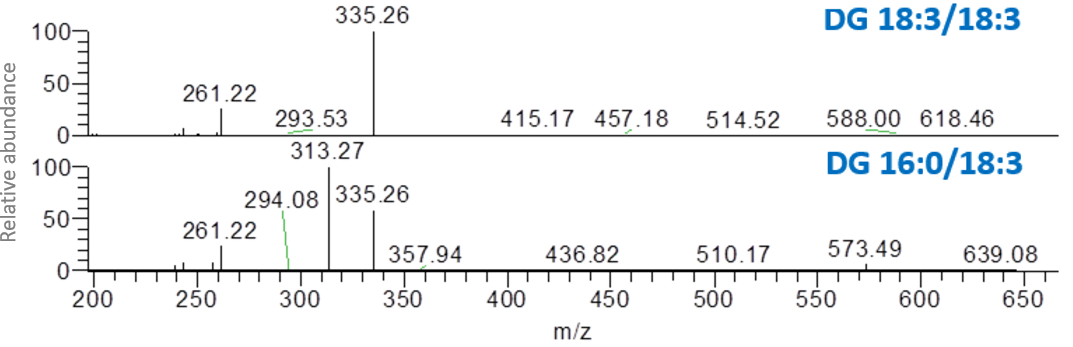


**Additional file 8: Fig. S6.** UHPLC-HRMS/MS mass spectra of DG 18:3/18:3 and DG16:0/18:3 which were unique biomarkers differentiating ORI vs. CRI.
