# Supplementary material for: Lipid mediated plant immunity in susceptible and tolerant soybean cultivars in response to Phytophthora sojae colonization and infection
Source: BMC Plant Biol. 2024 Mar 1;24:154. doi: 10.1186/s12870-024-04808-z (PMC10905861; doi:10.1186/s12870-024-04808-z)
Supplement: Supplementary file 9 — Supplementary Material 9. [file 12870_2024_4808_MOESM9_ESM.docx]

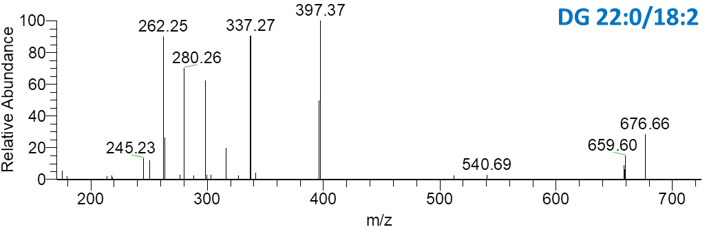


**Additional file 9: Fig. S7.** UHPLC-HRMS/MS mass spectrum of DG 22:0/18:2 which was the only unique biomarker differentiating OSC vs. OSI.
