# Supplementary material for: Lipid mediated plant immunity in susceptible and tolerant soybean cultivars in response to Phytophthora sojae colonization and infection
Source: BMC Plant Biol. 2024 Mar 1;24:154. doi: 10.1186/s12870-024-04808-z (PMC10905861; doi:10.1186/s12870-024-04808-z)
Supplement: Supplementary file 10 — Supplementary Material 10. [file 12870_2024_4808_MOESM10_ESM.docx]

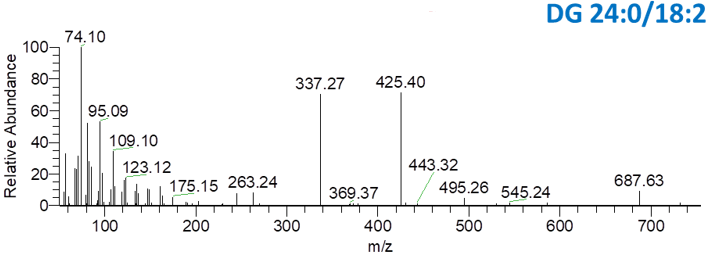


**Additional file 10: Fig. S8.** UHPLC-HRMS/MS mass spectrum of DG 24:0/18:2 which was one of the unique biomarkers differentiating OSI vs. CSI.
