# Supplementary material for: Lipid mediated plant immunity in susceptible and tolerant soybean cultivars in response to Phytophthora sojae colonization and infection
Source: BMC Plant Biol. 2024 Mar 1;24:154. doi: 10.1186/s12870-024-04808-z (PMC10905861; doi:10.1186/s12870-024-04808-z)
Supplement: Supplementary file 12 — Supplementary Material 12. [file 12870_2024_4808_MOESM12_ESM.docx]

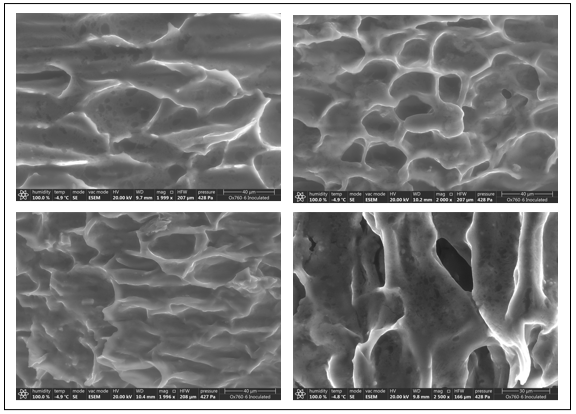


**Additional file 12: Fig. S10.** Scanning electron microscopy (SEM) demonstrating tiny opening of root cortical cells in the root tissue of soybean cultivars following inoculation.
