# Supplementary material for: Lipid mediated plant immunity in susceptible and tolerant soybean cultivars in response to Phytophthora sojae colonization and infection
Source: BMC Plant Biol. 2024 Mar 1;24:154. doi: 10.1186/s12870-024-04808-z (PMC10905861; doi:10.1186/s12870-024-04808-z)
Supplement: Supplementary file 14 — Supplementary Material 14. [file 12870_2024_4808_MOESM14_ESM.docx]

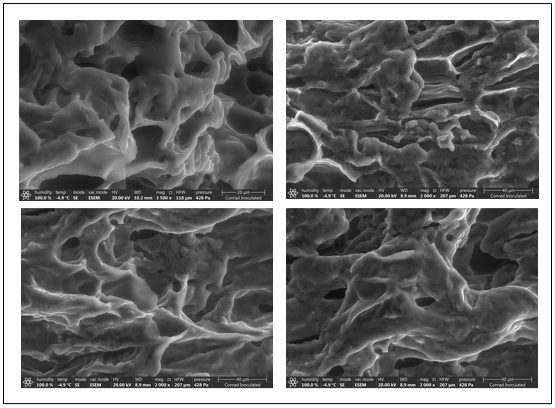


**Additional file 14: Fig. S12.** Scanning electron microscopy (SEM) demonstrating biogenic crystals in the root tissue of soybean cultivars following inoculation.
